# Enhancing Strain Capacity by the Introduction of Pearlite in Bainite and Polygonal Ferrite Dual-Phase Pipeline Steel

**DOI:** 10.3390/ma14185358

**Published:** 2021-09-17

**Authors:** Xingyang Tu, Yi Ren, Xianbo Shi, Changsheng Li, Wei Yan, Yiyin Shan, Ke Yang

**Affiliations:** 1 State Key Laboratory of Rolling and Automation, Northeastern University, Shenyang 110819, China; tut1810218@163.com; 2 State Key Laboratory of Metal Material for Marine Equipment and Application, Anshan 114009, China; 3 Institute of Metal Research, Chinese Academy of Sciences, Shenyang 110016, China; xbshi@imr.ac.cn (X.S.); weiyan@imr.ac.cn (W.Y.); yyshan@imr.ac.cn (Y.S.); kyang@imr.ac.cn (K.Y.)

**Keywords:** pipeline steel, microstructure, strain capacity, work hardening behavior

## Abstract

In this study the strain capacity and work-hardening behavior of bainite (B), bainite + polygonal ferrite (B + PF), and bainite + polygonal ferrite + pearlite (B + PF + P) microstructures are compared. The work hardening exponent (n), instantaneous work hardening value (n_i_), and differential Crussard-Jaoul (D_C-J_) analysis were used to analyze the deformation behavior. The best comprehensive mechanical properties were obtained by the introduction of the pearlite phase in B + PF dualphase with the tensile strength of 586 MPa and total elongation of 31.0%. The additional pearlite phase adjusted the strain distribution, which increased the initial work hardening exponent and then maintained the entire plastic deformation at a high level, thus delayed necking. The introduction of pearlite reduced the risk of micro-void initiation combined with the high frequency of high angle grain boundaries (HAGBs) in triple-phase steel, which led to a low crack propagation rate.

## 1. Introduction

Pipeline transport is considered the most economic, safe, uninterrupted, and large-scale method to transport oil and gas, geologically unstable regions become common in oil and gas transportation [1]. Since pipelines passing through these areas can undergo a high degree of plastic deformation, the development of pipeline steel with excellent deformability is crucial [2]. The pipeline material that has both high ultimate strain capacity and high ductile fracture resistance can help minimize damage caused by tension, compression, and bending loads.

Recently, the microstructure evolution, tensile properties, strain hardening, and microscopic deformation behavior of high-deformability pipeline steel have been reported [3,4]. Steels with a dual-phase microstructure containing soft and hard phases, usually exhibit better strain-hardening behavior than steels with single-phase microstructure. Pipeline steel with a polygonal ferrite and bainite (PF + B) microstructure has been widely investigated due to its high strength, toughness, and excellent deformability [5]. Tang et al. pointed out that the strain hardening exponent and uniform elongation first increased and then decreased with an increase in the bainite volume fraction and reached a peak value when the volume fraction of bainite was approximately 30% [6]. Zhao et al. reported that the bainite morphology influenced the strain capacity [7]. The distribution of banded bainite contributed to an improvement in the strength and plasticity compared to that of equiaxed bainite. Li et al. [8] and Cannmo et al. indicated that the difference in hardness between the hard and soft phase resulted in the formation of micro-void at the phase boundaries, which reduced the plasticity [9].

Several studies on improving the strain capacity of PF + B dual-phase steel have focused on the volume fraction, distribution, and morphological features of the ferrite and bainite phases. In general, the strain distribution during deformation determines the strain-hardening behavior and micro-void initiation mechanism prior to necking. The introduction of the second phase can improve the strain distribution [10,11], the strain distribution of dual-phase steel during tensile deformation is complicated and relates to the phase volume fraction, grain size, and mechanical properties. During the deformation process, the work-hardening behavior before necking varied due to the uneven strain distribution, and the better work-hardening properties of dual-phase steels helped delay the necking point [12]. After necking, the crack initiation and propagation mechanisms determine the subsequent deformation behavior. The cracks in multiphase steel mostly originate at the phase boundaries, which is mainly the result of local hardening due to the high density of geometrically necessary dislocations (GNDs) at the phase boundaries, as confirmed in ferrite and martensite (F + M) dual-phase steel [13]. 

Previous studies have only investigated the strain capacity of dual-phase pipeline steel; therefore, there is a research gap in the investigation of multiphase microstructures. There is no clear consensus on the effect of introducing the third microstructure component into the dual-phase on the mechanical properties. Compared with F + M and F + B dual-phase steels, Sudo et al. [14] have indicated that the F + B + M triple phase steel had the best combination of high strain hardenability (high n value), shape fixability (low yield ratio), and stretch-flangeability. However, Kim et al. pointed out the triple-phase (F + M + B) had inferior mechanical properties compared to conventional steel [15]. Ghorabaei et al. tried to introduce pearlite (P) into F + M dual-phase steel and found the introduction of pearlite can greatly enhance the deformability [16]. The possible excellence deformability is the development goal of high deformation pipeline steel; therefore, it is interesting to further study the structure-property relationships of triple-phase pipeline steel. This design idea may become an important method to improve deformability of pipeline steel.

In this study, single phase (B), dual-phase (B + PF) and triple-phase (B + PF + P) microstructures with the same alloy components were obtained by varying the thermomechanical control process (TMCP). The strain capacity, micro-void initiation mechanism, and microcrack propagation behavior were investigated by tensile tests. The results and related discussion will provide an important basis for improving the strain capacity of high-deformability pipeline steel.

## 2. Experimental

### 2.1. Materials

**Figure 1:**
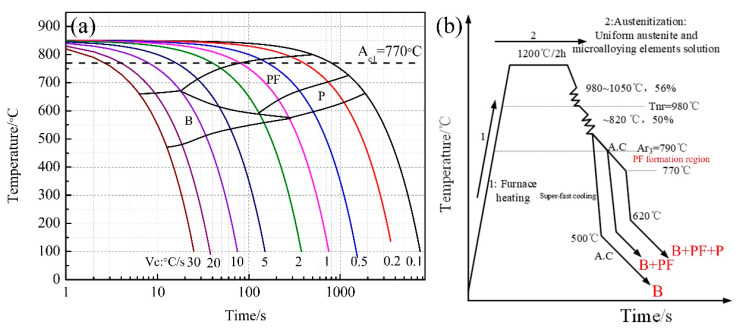
(**a**) DCCT curve, (**b**) Schematic of thermomechanical control process (TMCP).

A low-carbon micro-alloyed test steel was produced in the laboratory; the chemical composition is shown in Table 1. The equilibrium transformation temperatures Ar_1_ and Ar_3_ were determined using a DIL805A/D as 660 °C and 790 °C, respectively; the parameters of TMCP (Figure 1b) are formulated using deformation continuous cooling transformations (DCCT) diagram (Figure 1a). The non-recrystallization temperature was calculated using Equation (1) as 980 °C [17].

(1) $$T_{\mathrm{nr}}({}^{\circ}C)=887+464C+890\mathrm{Ti}+363\mathrm{Al}-357\mathrm{Si}+\left(6445\mathrm{Nb}-644\sqrt{\mathrm{Nb}}\right)+\left(732V-230\sqrt{V}\right)$$

Three blocks with dimensions of 80 mm × 70 mm × 65 mm were reheated to 1200 °C and soaked for 2 h. Rough rolling was performed within the austenite recrystallization region to reduce the thickness from 80 to 35 mm. The finishing rolling temperature ended at 820 °C, and the thickness of the plates was ~15 mm. Steel with a bainite microstructure was obtained by direct super-fast cooling after rolling. Two other steels with different microstructures, identified as B + PF and B + PF + P steels, were obtained by adjusting the cooling route. B + PF and B + PF + P steels were air-cooled to temperatures below A_r3_ (790 and 770 °C, respectively) after rolling was complete. The relaxation process in the ferrite-austenite dual-phase region can induce a certain amount of polygonal ferrite formation; therefore, the cooling rate was controlled by a super-fast cooling process. The cooling rate was above 30 °C/s, and the cooling stop temperature were ~500 °C and ~620 °C, respectively. Finally, coil cooling for all steel blocks was simulated by air cooling to room temperature. 

### 2.2. Microstructural Characterization and Mechanical Property Test

Microstructures were observed using optical microscopy (OM) (LEICA DMIRM, Jena, Germany), scanning electron microscopy (SEM) (ZEISS Ultra 55, Hallbergmoos, Germany), and electron backscatter diffraction (EBSD) (ZEISS Ultra 55, Jena, Germany). Samples taken from transverse cross-sectional plates of steel were mechanically ground, polished, and etched using a 4% Nital solution. The samples were electropolished using an electro-polisher for EBSD analysis. EBSD scanning was performed using a TSL system installed on a Zeiss ULTRA 55 FE-SEM with a step size of 0.23 μm and accelerating voltage of 20 kV. The EBSD data were analyzed using Channel 5 software (Oxford Instruments plc, Abingdon, UK). The phase composition was measured using a D/max2400 X-ray diffractometer (XRD) (Shimadzu, Kyoto, Japan) with a 2θ range from 42° to 102°, and the scanning speed was 1°/min. The tensile test was conducted on a SANS-CMT 5205 tensile tester (CMT 5205, Kexin, Changchun, China) at room temperature with a cross beam at a speed of 1 mm/min (8.310^−4^ s^−1^). Specimens with a diameter of 3 mm and gauge length of 30 mm were machined along the rolling direction according to GB/T 228.1-2010 standard [18]. Two tensile tests were performed on each microstructure to ensure repeatability. The fracture surface and surfaces perpendicular to the tensile fracture were observed by SEM.

### 2.3. Work-Hardening Behavior Analysis

The work-hardening behaviors were analyzed using the following Hollomon equation [19]:

(2) $$\sigma ={K\varepsilon }^{n}$$

where σ and ε are the true stress and true strain, respectively, K is the strength coefficient, and n is the work-hardening exponent. The Hollomon equation can also be expressed as lnσ=lnK+nlnε; the instantaneous work-hardening exponent can be defined as $n_{i}=\left(\frac{\varepsilon }{\sigma }\right)\left(\frac{d\sigma }{d\varepsilon }\right)$ [20]. The instantaneous work-hardening exponent $n_{i}$ has been widely used as an indication of steel formability. The relationship of lnσ−lnε also was considered, where $\frac{d\sigma }{d\varepsilon }$ can be defined as the work-hardening rate, in addition, $\frac{d\sigma }{d\varepsilon }=\sigma$ with $\varepsilon =\varepsilon_{u}$, is defined as the criterion for necking, where $\varepsilon_{u}$ is the maximum value of uniform elongation. The plot of $\ln(\frac{d\sigma }{d\varepsilon })$ vs. lnε can be used to study the relationship between the work-hardening behavior [21] and differential Crussard-Jaoul (D_C-J_) analysis based on the Ludwik equation.

(3) $$\mathrm{Ludwik}\Rightarrow \sigma =\sigma_{0}+K_{S}\varepsilon^{n_{S}}\to \ln\frac{d\sigma }{d\varepsilon }=\ln(K_{S}n_{S})+\left(n_{S}-1\right)\ln\varepsilon$$

where $\sigma_{0}$ and K_s_ are constants, and n_s_ is the inverse of the work-hardening exponent.

## 3. Results

### 3.1. Microstructures 

**Figure 2:**
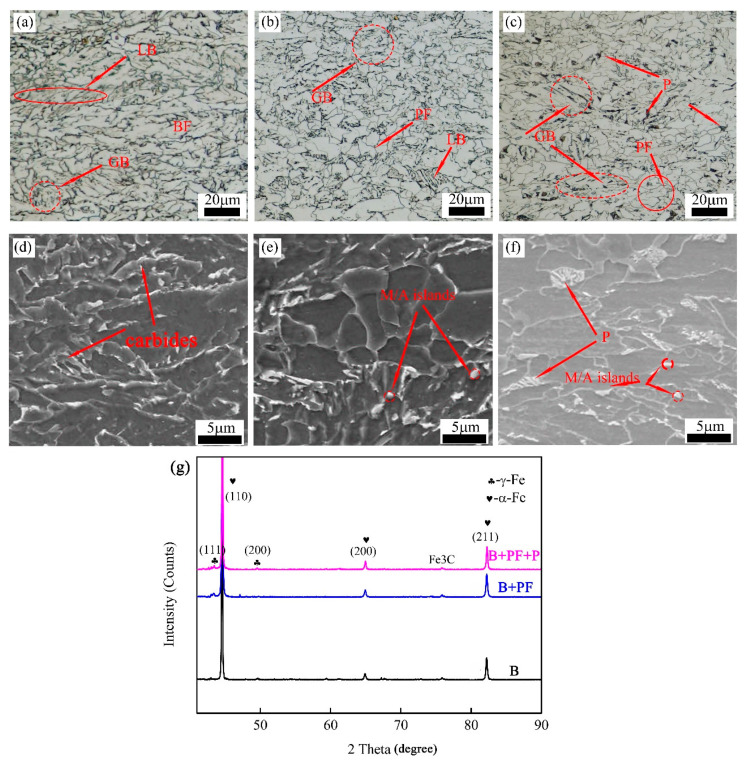
(**a**,**d**) Single-phase (B); (**b**,**e**) dual-phase (B + PF); (**c**,**f**) triple-phase (B + PF + P); and (**g**) XRD analysis results.

The single-phase (B), dual-phase (B + PF), and triple-phase (B+ PF+ P) microstructures are shown in Figure 2. The XRD analysis results (Figure 2d) indicated the phase compositions of the three samples are α-ferrite, retained austenite (RA) and carbides (cementite), and that the low volume fraction of RA led to (111)γ and (200)γ crystal plane diffraction peaks almost unobservable. Single-phase is mainly composed of granular bainite (GB), lath bainite (LB), and bainite ferrite (BF) (Figure 2a). Many carbides were found in the bainite matrix, as shown in Figure 2d. When the initial cooling temperature decreased to 790 °C, a dual-phase microstructure was obtained (Figure 2b,e). Compared with the single-phase microstructure, the volume fraction of the LB phase decreased, and the volume fraction of PF was about approximately 34%. Moreover, many martensite and austenite (M/A) islands (marked by red arrows in Figure 2e) were observed at the BF boundaries. When the initial cooling temperature decreased to 770 °C, the longer relaxation process led to an increase in the volume fraction of PF (38%), and the pearlite phase was observed [22,23], as shown in Figure 2c,f. The higher volume fraction of PF promotes the stability of the austenite, and the formation rich and poor carbon zones in austenite provides conditions for the formation of pearlite.

**Figure 3:**
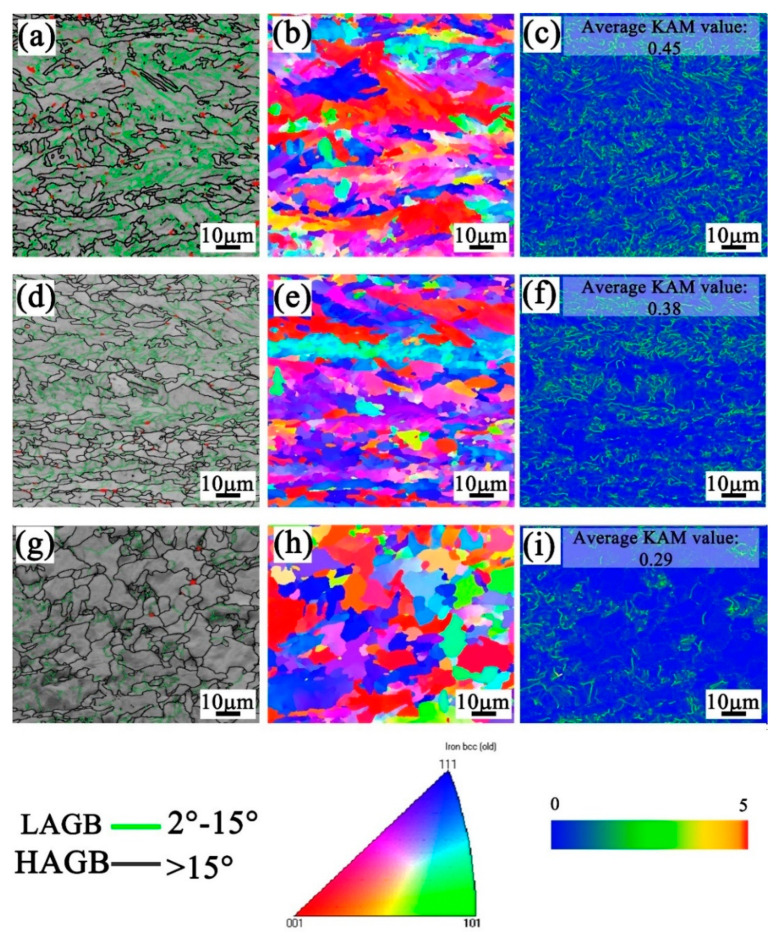
IQ images, IPF figures, and KAM maps: (**a**–**c**) single phase, (**d**–**f**) dual-phase, (**g**–**i**) triple-phase.

**Figure 4:**
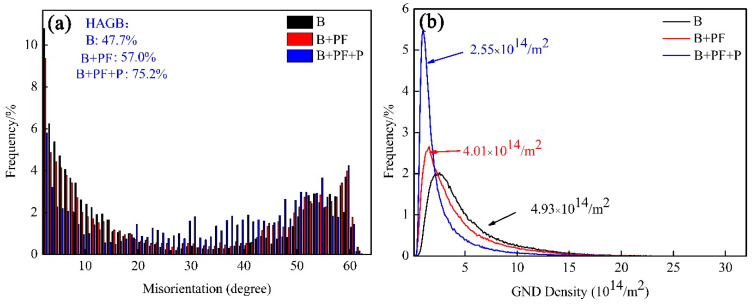
(**a**) The frequency of HAGBs and (**b**) GND density.

Image quality (IQ) image, inverse pole figure (IPF), and kernel average misorientation (KAM) map of three samples were obtained from EBSD data and are shown in Figure 3. For IQ images (Figure 3a,d,g), the high angle grain boundaries (HAGBs) and low angle grain boundaries (LAGBs) were depicted by black lines and green lines, respectively, whereas the retained austenite (RA) phases were depicted by red pixels. The frequency of HAGB for three samples is shown in Figure 4a. The frequency of HAGB increased with an increase in the phase number; the triple-phase microstructure showed the highest HAGB frequency. The RA content of the triple-phase microstructure decreased to a minimum, which is caused by the formation of the pearlite phase. In Figure 3b,e,h, the different colors indicate the crystallographic orientation aligned with the normal surface orientation of the samples. Bainite showed much higher orientation gradients than the polygonal ferrite; the orientation of the samples became random as relaxation process increased. Furthermore, the KAM maps (Figure 3c,f,i) show that the strain is mainly distributed in the bainite and pearlite phase with large lattice distortion; the average KAM value decreased with the introduction of polygonal ferrite and pearlite phases. The geometrically necessary dislocation (GND) density can be estimated with KAM values from Equation (4) [24]:

(4) $$\rho_{{GND}_{s}}=\frac{2\theta }{\mu b}$$

where μis the unit length, *b* is Burgers vector, and θ is the misorientation angle. As shown in Figure 4b, the density of GNDs in single-phase, dual-phase, and triple-phase samples is 4.93 × 10^14^/m^2^, 4.01 × 10^14^/m^2^, and 2.55 × 10^14^/m^2^, respectively. The triple-phase sample had the lowest value.

### 3.2. Tensile Properties

**Figure 5:**
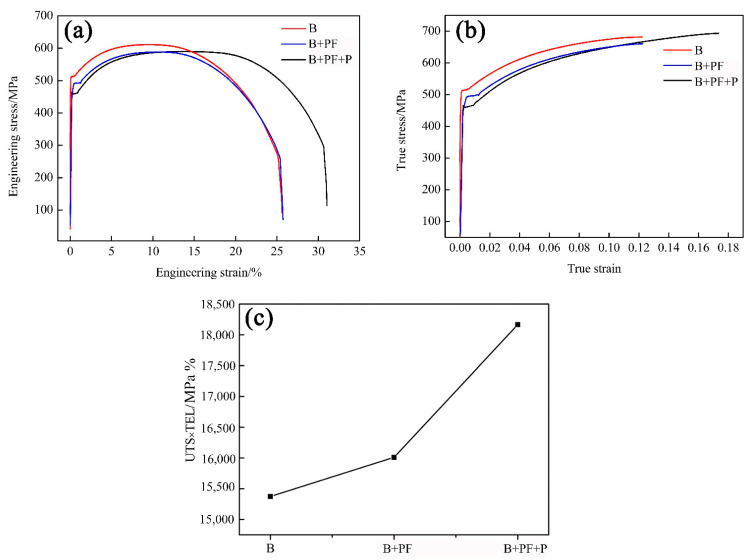
(**a**) Engineering stress-strain curves (**b**) true stress-strain curves, and (**c**) PSE.

The engineering and true stress-strain curves of tested samples are presented in Figure 5a,b. The tensile properties are summarized in Table 2. All the samples demonstrated the yield point phenomenon, which is believed to be related to the Cottrell atmospheres due to interstitial atoms around dislocation [25]. The yield strength of the triple-phase sample was lower than that of the others, but the elongation was noticeably higher. The single-phase sample showed the highest yield and tensile strength, and the total elongation was nearly the same as that of the dual-phase sample. The tensile strengths of the dual-phase and triple-phase samples were nearly the same. As Figure 5b shows, the true stress-strain curves present the same characteristics as the engineering stress-strain curves prior to the necking point. The ultimate tensile strength (UTS) elongation and total elongation (TEL) of the three samples are different; the triple-phase sample showed the maximum value, followed by the dual-phase sample, while the single-phase sample had the minimum value. The UTS × TEL (PSE) values are shown in Figure 5c to compare the comprehensive mechanical properties. The largest PSE value (18,166% MPa) was obtained from the triple-phase sample, followed by dual-phase sample, and the single-phase sample which showed the minimum value. 

### 3.3. Work Hardening Behavior

**Figure 6:**
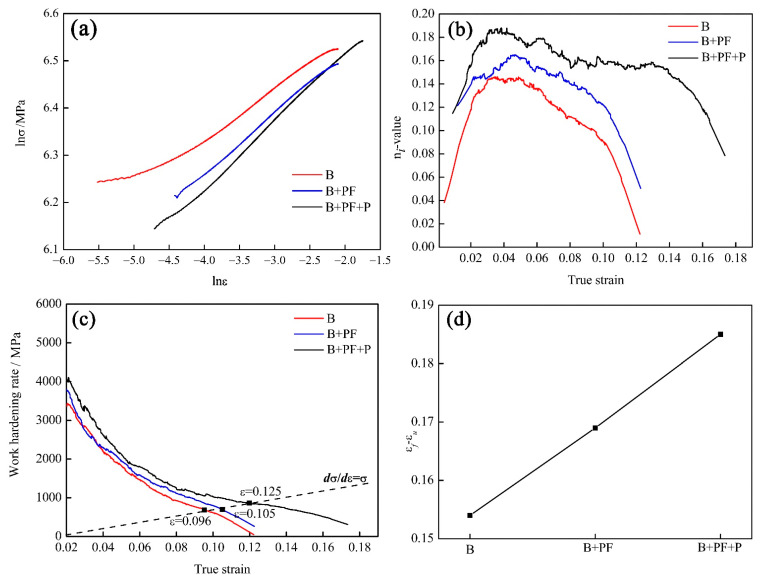
(**a**) lnσ−lnε curves, (**b**) n_i_ values, (**c**) work-hardening rate (**d**) extended strain after necking.

The stress-strain curves for different microstructures were analyzed using the Hollomon method, as shown in Figure 6. The lnσ−lnε curves failed to show linearity in the entire uniform strain stage (Figure 6a). The work-hardening parameters, namely, the *n* and *K* values, are summarized in Table 3 and Table 4. For the three samples, the maximum *n* value increased with the introduction of polygonal ferrite and pearlite; the maximum *K* value showed the same trend. In addition, the *n* and *K* values of the single and triple-phase samples increased initially and then decreased as the strain increased. The *n* and *K* values of dual-phase sample show a decreasing trend with increasing strain [26]. 

To further analyze the variation in the n-value at different strain stages, the relationship between the instantaneous work-hardening exponent (n_i_) and true strain was analyzed. The triple-phase sample has the highest n*_i_* value over the entire strain stage, while the n*_i_* value of others decreased as the phase number decreased (Figure 6b). It must be clearly distinguished from the n*_i_* value with the work-hardening rate. In this study, the higher n*_i_* had a higher work-hardening rate when the true strain was over 0.015, as shown in Figure 6c and Table 3. Furthermore, the extended strain after necking of the three samples showed an upward trend as the phase number increased. 

### 3.4. Differential C-J Analysis

**Figure 7:**
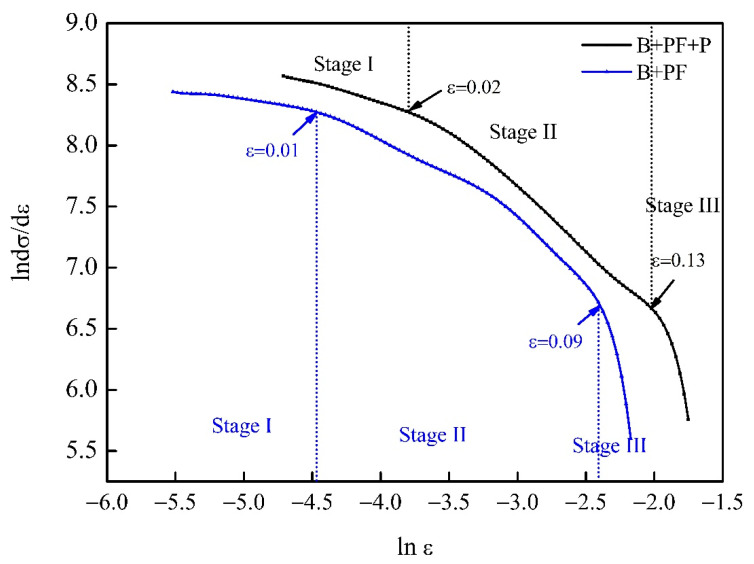
D_C-J_ plots of ln(dσ/dε) versus ln (ε) for dual-phase and triple-phase sample.

The D_C-J_ analysis curves of the dual-phase and triple-phase samples are shown in Figure 7. The different slopes indicate different cooperative deformation behaviors, and the strengthening mechanism of different steels depends on the slope of the line segment in the D_C-J_ curve. A rapid decreased in the work-hardening rate with an increase in strain or a high rate of dynamic recovery leads to a lower slope. Conversely, a larger slope indicates a relatively slow decrease in the work-hardening rate [27]. In this study, three stages are observed in the D_C-J_ curves for the dual-phase and triple-phase samples, and the transition strains of each stage are marked in Figure 7. The transition strains from stage I to stage II and stage II to stage III of the dual-phase sample were lower than those of the triple-phase sample.

### 3.5. Fracture Morphology 

**Figure 8:**
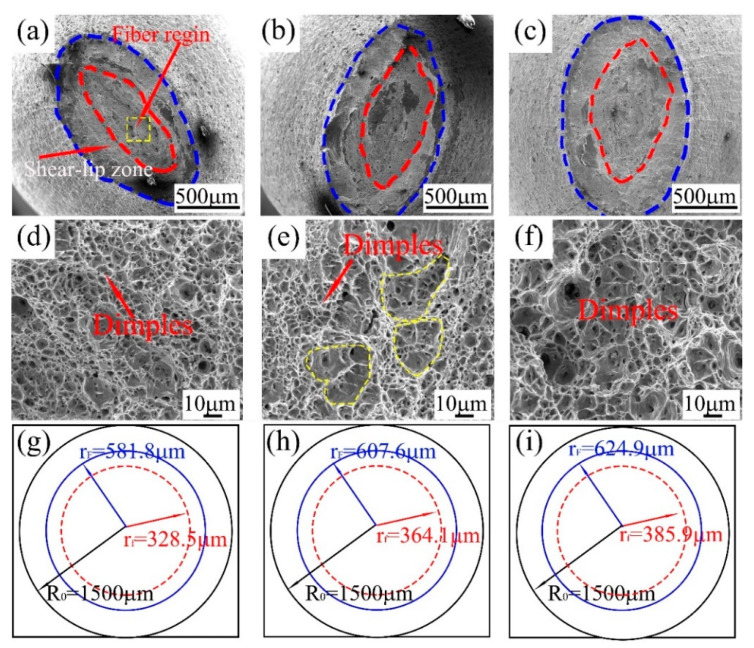
Tensile macro fractography images, microstructures of the fiber zone, and schematic diagram of different fracture zones: (**a**,**d**,**g**) single phase, (**b**,**e**,**h**) dual-phase, (**c**,**f**,**i**) triple-phase.

The tensile fractography images of the three samples are shown in Figure 8, ductile fractures with fiber and shear-lip zones can be observed in all fracture surfaces (Figure 8a#x2013;c). The area of the fiber zone increased with an increase in the phase number; in addition, numerous dimples were observed in the fiber zone, and both the size and depth of the dimples increased with an increase in the phase number (Figure 8d–f). The large dimples (marked by the yellow dotted line) in the dual-phase sample were caused by the severe plastic deformation of the polygonal ferrite distributed in the matrix. 

The schematic with different circles of the fracture microstructures represents different fracture zones, as shown in Figure 8g,h,i. Mao et al. reported that the relationship between the amount of inhomogeneous plastic deformation and the crack propagation rate can be described as follows [28]:

(5) $$v=\left[r_{f}\cdot \dot{\varepsilon }/\left(R_{0}-r_{F}\right)\right]/{\Delta \varepsilon }_{T}$$

where v indicates the crack propagation rate (μm/s), $r_{f}$ indicates the average value of the fiber zone (μm), and $r_{F}$ represents the sum value of the fiber and shear-slip zone (μm). Further, the $\dot{\varepsilon }$ = 8.3 × 10^−4^ s^−1^, R_0_ = 1500 μm, ${\Delta \varepsilon }_{T}$ is shown in Figure 6d. 

**Figure 9:**
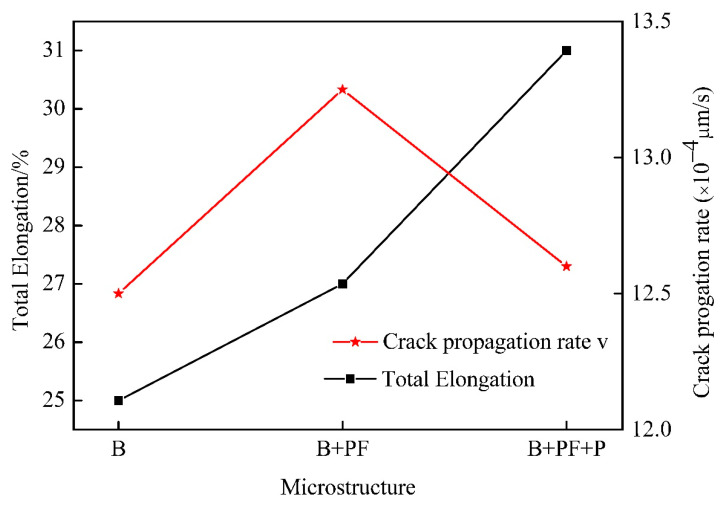
Variations in elongation versus fracture and crack propagation rate for different samples.

Figure 9 shows the relationship between the crack propagation rate and total elongation. The crack propagation rate of single-phase sample was much higher than that of dual-phase and triple-phase samples; thus, the single-phase sample showed the lowest total elongation.

**Figure 10:**
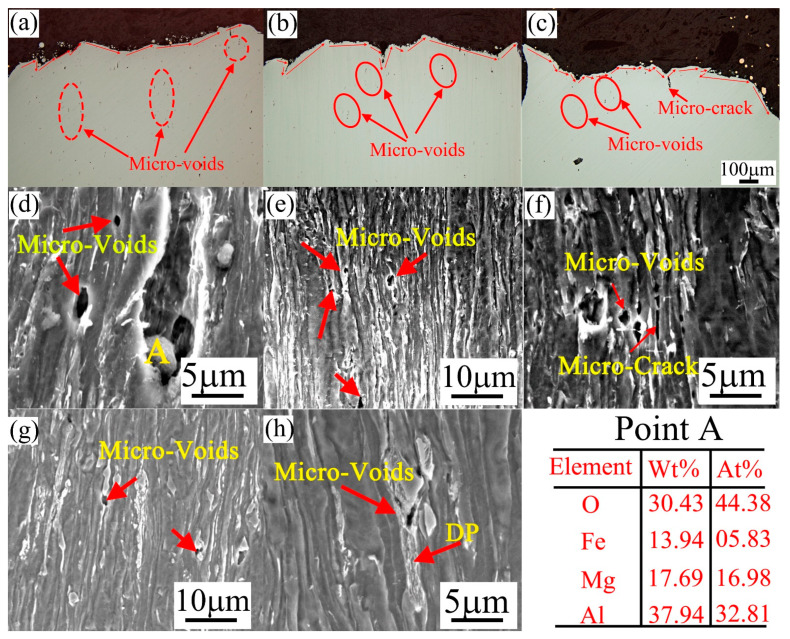
OM morphologies of the surfaces perpendicular to the tensile facture: (**a**) single-phase, (**b**) dual-phase, (**c**) triple-phase; SEM microstructures: (**d**–**f**) dual-phase, (**g**,**h**) triple-phase.

The fracture morphologies parallel to the tensile direction are shown in Figure 10. The main crack propagation paths of all the samples are marked with red lines. The more tortuous the crack propagation paths, the higher the crack propagation work required during the propagation process [29]. As shown in Figure 10c, the more tortuous crack propagation paths of the triple-phase sample indicated that it can withstand large strain to fracture, which is consistent with its high elongation. The density of micro-voids decreased significantly in the dual-phase and triple-phase samples. The microcracks shown in Figure 10b,c, are caused by Mg/Al oxide inclusions during the smelting process (EDS results of pint A of Figure 10d). Ohata et al. found that the voids originating from large inclusions cannot cause ductile damage in the dual-phase steel [30]. Instead, it was caused by the formation of a particular number of voids near the boundaries of the hard-soft phase. As shown in Figure 10e,f, the location of the micro-void in bainite was mainly located at the BF/BF, BF/carbides boundaries. However, the ferrite and pearlite in the triple-phase sample can elongate along the tensile direction (Figure 10g,h), and the micro-voids mainly formed at the boundaries of the ferrite and M/A island, and were more distributed. 

## 4. Discussion

The single-phase, dual-phase, and triple-phase samples were obtained using different TMCP parameters. The mechanical properties indicated that the introduction of pearlite led to a decrease in the yield strength but an increase in strain capacity. The carbon content in the bainite for the dual-phase sample must be higher than that in triple-phase sample; thus, the hardness of bainite in triple-phase sample was lower than that in dual-phase sample. This is confirmed by the lower density of GNDs in the triple-phase steel. Luis et al. found that the strength of the mixed phase steel increased linearly with an increase in the hard phase (martensite or bainite) [31]. Furthermore, an increase in the hard phase directly contributes to the ultimate strength, yield strength, and ductility. Thus, the triple-phase sample exhibited a lower yield strength and higher ductility. In this study, the tensile strength of triple-phase and dual-phase samples were almost the same. The work-hardening behavior in the plastic deformation stage played an important role in improving the strength and plasticity, while the continuous work-hardening of the polygonal ferrite phase to full deformation without failure was the main reason for the high tensile strength, which is determined primarily by the coordination deformation ability of each phase in the triple-phase sample. 

The microstructural deformation can be determined from the D_C-J_ analysis. There are three deformation stages in multiple-phase steel that are associated with the following deformation mechanism:

1. Stage I: Deformation of soft ferrite matrix;
2. Stage II: Simultaneous deformation of soft and hard phases;
3. Stage III: Dynamic recovery in ferrite.

The introduction of pearlite in the dual-phase sample delayed the transition strain of each stage and changed the work-hardening mechanism. Work-hardening ability is one of the most important characteristics for industry production. The formability and plasticity of materials were determined by their work-hardening ability [32]. The effect of phase composition on the mechanical properties of pipeline steel indicated that the optimum work-hardening capacity could be obtained with triple-phase sample. A trade-off existed between the total elongation and tensile strength values, and the work-hardening ability was at an equilibrium stage (Table 2). The dual-phase sample showed better work-hardening ability and mechanical properties than the single-phase sample. It is common that the inverse proportion of strength to plastic in pipeline steel, results in these opinions. The loss of strength can be complemented by an increase in total elongation. The work-hardening behavior before the necking stage and the damage mechanism after the necking stage determine the total elongation. 

The necking point was determined using the necking criterion ($\frac{d\sigma }{d\varepsilon }=\sigma$), which means that the uniform deformation ended. and the necking occurred at this point (Figure 6c). This indicated that the high work-hardening rate of triple-phase sample resulted in a large uniform elongation. The higher the n*_i_* value, the higher the work-hardening rate, and formable materials exhibited higher n*_i_* values to maintain a high strain level [33]. As shown in Figure 6c, the n*_i_* of the three samples increased rapidly during the initial stage of uniform deformation, and the initial n*_i_* increased with an increase in the ferrite volume fraction. The hardening effect of dislocation entanglement in the bainite phase was stronger than the nonplanar dislocation structure slip and cell structure formation in ferrite during tensile deformation [34], and the decrease in the strain-hardening rate in bainite induced the n*_i_* to rapidly increase to its maximum value (Figure 6b). In addition, the peak value of triple-phase sample was much higher than that of others, and the higher work-hardening behavior was sustained to the UTS, with the deformation stability of triple-phase sample playing an important role. Compared with the triple-phase sample, the n*_i_* of the single-phase and the dual-phase samples immediately decreased when n*_i_* reached the peak value. The optimum work-hardening ability of the triple-phase sample contributed to the larger deformation behavior. 

In essence, bainite is a complex structure of α-ferrite and carbides. Park et al. also showed that replacing carbides with a second phase (e.g., martensite) resulted in a significant increase in the strain-hardening rate [35]. For dual-phase steel, significant internal stresses were primarily transferred into the polygonal ferrite as the carbon-rich austenite transformed to bainite, and the residual stress was produced simultaneously [36]. Many GNDs can be generated in polygonal ferrite near the phase boundaries, leading to local hardening of the polygonal ferrite [37]. The residual stress is eliminated at the initial plastic deformation, and the dislocation and back stress (induced by incompatibility phase transformation of bainite and polygonal ferrite phases) in polygonal ferrite can contribute to an increase in the strain hardening rate. 

Researchers have found that the RA can induce martensitic transformation by strain, which names transformation-induced plasticity (TRIP) effects for improving deformation ability and ductility [38]. The TRIP effect depends on the amount of retained austenite and its stability to deformation induced transformation. Many researchers have indicated that the obvious TRIP effect only occurred when the retained content of RA exceeds 5% [39,40,41], and the stability of RA is associated with its carbon content [42]. In this study, the RA content of all tested samples were lower than 5%, besides, the formation carbides in the pearlite phase which decreased the carbon content in RA to reduce its stability. Thus, there were no obvious TRIP effects on the stress-strain curves because of the low fraction and stability of RA in the matrix inhibited these effects.

The hardening mechanism was enhanced by the addition of a pearlite phase when compared with the hardening mechanism of the dual-phase sample. The lamellar structure of pearlite is composed of deformable ferrite and hard-brittle cementite [43]. The interface between the ferrite and carbide lamella is the position where dislocations are generated in ferrite [44]. During the phase transformation process, both the soft ferrite in the pearlite and the polygonal ferrite were affected by the compressive stress formed by the transformation of the surrounding austenite to bainite. The deformation of ferrite in pearlite was also suppressed by cementite; dislocations (GNDs) were formed at the interface of cementite and ferrite which further hardened the pearlite. Under this mechanism, the density of GNDs at the interface between polygonal ferrite and bainite or pearlite is significantly lower than that of bainite–ferrite–cementite, and the dislocation accumulation increased the shearing force of the dislocation tip zone, which induced localized plastic flow at the ferrite/carbides interface under low stress [45] resulted in the continuous strain-hardening of pearlite during deformation. The elongated pearlite morphology without micro-void formation, as shown in Figure 10g,h, which indicated that the pearlite in the triple-phase sample had optimum hardening behavior, and the work-hardening ability was adjusted.

The damage mechanism after the necking stage also needs to be considered. The elongation after necking of the multiphase steels was much higher than that of the single-phase sample (Figure 6d). The damage mechanism after necking mainly includes three processes: micro-void initiation, crack propagation, and fracture [46]. The results showed that multiphase steel can effectively inhibit plastic damage and promote a significant increase in elongation after necking. The fracture morphology indicated that the micro-void was mainly concentrated at the interface of the M/A islands (or carbides) and soft ferrite, and the introduction of pearlite reduced the risk of micro-void initiation and combination owing to the better work-hardening behavior and more certain deformability. In addition, it is well known that HAGBs with high grain boundary energy can effectively hinder dislocation motion and crack propagation [47], the high frequency of HAGBs in triple-phase sample led to a low crack propagation rate. 

## 5. Conclusions

(1) A triple-phase steel with B + PF + P microstructure was produced by the TMCP process. The lower final relaxation temperature contributed to form a higher volume fraction of PF led to the carbon enriched in austenite; the forming rich and poor carbon zone provides conditions for the formation of pearlite. 

(2) The best comprehensive mechanical properties can be obtained by the introduction of pearlite. The tensile strength and total elongation can reach to 560 MPa and 31%, the largest elongation was attributed to the addition of the pearlite.

(3) The pearlite exhibited as a ductility phase due to the optimum strain-hardening behavior in triple-phase steel, the continuous strain-hardening of pearlite can adjust the coordination deformation to delay the strain of soft phase transfer to hard phase (D_C-J_ curve transfer strain). Besides, the crack initiation points can be reduced by the introduction of pearlite phase, combination with the large frequency of HAGBs, which can hind the crack propagation during tensile deformation contributed to the excellent work hardening behavior and the lowest crack propagation rate. 

## Figures and Tables

**Table 1:** Chemical composition of the experimental steel (wt.%).

C	Si	Mn	P	S	Al	N	Cr	Cu + Ni	Nb + V + Ti + Mo
0.06	0.22	1.6	0.012	0.002	0.03	0.007	0.11	0.02	0.126

**Table 2:** Mechanical properties of three microstructures.

Microstructure	Yield Strength/MPa	Ultimate Tensile Strength (UTS)/MPa	Yield Ratio	Work-Hardening Exponent(n)	Total Elongation (TEL)/%
B	513	615	0.83	0.09	25.0
B + PF	486	593	0.82	0.10	27.0
B + PF + P	455	588	0.77	0.12	31.0

**Table 3:** The work hardening exponent of three samples.

Sample	Work Hardening Exponent, n			
	0.5–1.5%	1–6%	5–12%	10–20%
Single-phase (B)	0.05	0.11	0.09	0.05
Dual phase (B + PF)	0.13	0.13	0.11	0.08
Triple-phase (B + PF + P)	0.1	0.15	0.14	0.12

**Table 4:** The strength coefficient of three samples.

Sample	Strength Coefficient, K/MPa			
	0.5–1.5%	1–6%	5–12%	10–20%
Single-phase (B)	691	869	841	760
Dual phase (B + PF)	890	884	849	780
Triple-phase (B + PF + P)	746	907	897	866

## Data Availability

Not applicable.
